# Optimization of heterogeneous vehicle logistics scheduling with multi-objectives and multi-centers

**DOI:** 10.1038/s41598-023-41450-5

**Published:** 2023-08-29

**Authors:** Zhaolei He, Miaohan Zhang, Qiyong Chen, Shiyun Chen, Nan Pan

**Affiliations:** 1 Metrology Center, Yunnan Power Grid Co., Ltd, Kunming, 650041 People’s Republic of China; 2 Faculty of Civil Aviation and Aeronautics, Kunming University of Science and Technology, Kunming, 650500 People’s Republic of China

**Keywords:** Applied mathematics, Computational science, Information technology

## Abstract

Industrial enterprises have high requirements on timeliness and cost when delivering industrial products to their customers. For this reason, this paper studies the vehicle routing problem (VRP) of different vehicle models in multiple distribution centers. First of all, we consider the multi-dimensional constraints in the actual distribution process such as vehicle load and time window, and build a multi-objective optimization model for product distribution with the goal of minimizing the distribution time and cost and maximizing the loading rate of vehicles. Furthermore, an Improved Life-cycle Swarm Optimization (ILSO) algorithm is proposed based on the life cycle theory. Finally, we use the order data that Yunnan Power Grid Company needs to deliver to the customer (municipal power supply bureau) on a certain day to conduct a dispatching experiment. The simulation and application results show that the transportation cost of transportation obtained by the ILSO algorithm is reduced by 0.8% to 1.6% compared with the other five algorithms. Therefore, ILSO algorithm has advantages in helping enterprises reduce costs and improve efficiency.

## Introduction

**Figure 1:**
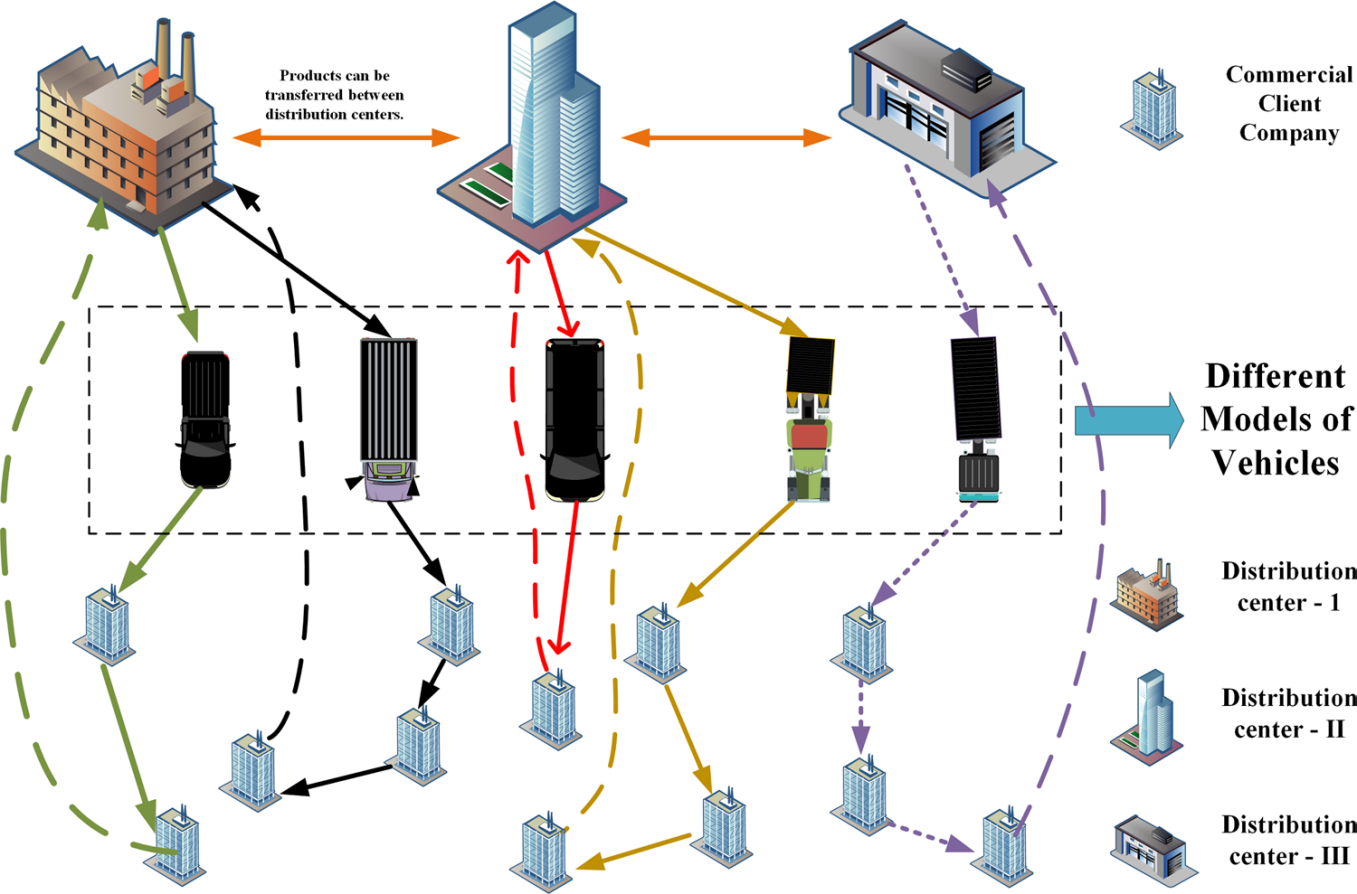
Business model flow chart.

The Council of Supply Chain Management Professionals (CSCMP) in its "33rd annual state of logistics report" shows that although the inventory of American commercial logistics enterprises fell to the lowest level in history in 2021, the related transportation costs increased by 21.7%^1^. Therefore, many enterprises urgently need to reduce transportation costs. At present, the loading rate of trucks used by the Metrology Center of China Southern Power Grid Co., Ltd to distribute electric power metering devices (a kind of electric power equipment) to the municipal power supply bureau is only about 55%, which wastes a lot of transportation resources^2^. In view of the current problems in the electric power equipment logistics distribution system of power enterprises, such as low quality and efficiency of decision-making, unscientific and unreasonable scheduling^2^, this paper studies the corresponding VRP problem against the background of the electric power equipment logistics distribution of Yunnan Power Grid Corporation, so as to successfully help power enterprises achieve cost reduction and efficiency increase. The logistics distribution of electric power equipment can be summed up as the problem of highway trunk transportation in cargo transportation. The core of trunk transportation is to solve the problem of trunk transportation no-load rate^3^, that is, to increase the quantity of electric power equipment delivered by a single vehicle on the premise of ensuring the delivery time. Below, we briefly describe the specific characterization of the problem, in which the business model flowchart is shown in Fig. 1.

For power grid enterprises, there are usually multiple distribution centers that deliver goods to the municipal power supply bureau within their jurisdiction, and the models of transportation vehicles in the distribution centers are different. For example, China Southern Power Grid Co., Ltd has four logistics distribution centers in different cities. Its customers (municipal power supply bureau) are also distributed in different cities, and each logistics distribution center has different transport vehicles to distribute electric power equipment. In order to maintain profitability, the transportation companies only carry out the distribution task when the actual load tonnage of vehicles is higher than the minimum load limit. In addition, it is also necessary to consider the time window limit of the arrival time of electric power equipment required by each municipal power supply bureau. Therefore, the distribution of electric power equipment has the characteristics of multiple vehicle types, covering multiple cities, and multiple distribution centers. It is a multi-objective complex vehicle routing problem (VRP) involving many constraints^4,5^.

In view of the above problems, different from the existing research, this paper constructs a multi-objective optimization model involving power equipment distribution. In the case of considering more real constraints, the minimum fitness function of distribution cost, the minimum fitness function of delivery time and the maximum fitness function of the municipal power supply bureau for single vehicle distribution are built, which makes the established mathematical model more practical. Further, we developed a heuristic algorithm, which draws on the biological life cycle characteristics to improve the group search optimization algorithm (GSO). Then, the variant group strategy is introduced to design an improved life-cycle swarm optimization (ILSO) algorithm for the model solution. Finally, based on the actual business scenario data, the simulation experiments are compared with other heuristic algorithms.

The paper is organized as follows: in Section “Literature review”, we present the existing literature studies. In Section “Modeling the distribution path problem of heterogeneous vehicles for industrial products in multiple-depot with time windows”, we construct the corresponding mathematical model. In Section “Model solution based on ILSO algorithm”, we design the ILSO algorithm. In Section “Results and analysis”, we show the simulation results with the application of the model and algorithm. Finally, in Section “Conclusion”, we summarize the whole paper.

## Literature review

The VRP problem was first proposed by Dantzig et al. in 1959 in order to solve the distribution problem of oil tank trucks^6^. Further, Clarke et al. proposed a heuristic algorithm called C-W saving algorithm to solve VRP problem^7^. On this basis, relevant researchers have carried out extensive research on the problem, improved relevant models and algorithms, and studied the variants of VRP^8,9^. Therefore, this paper reviews the problem from three aspects: relevant models, algorithms and variants of the problem.

### Model

At this stage, most of the research on logistics distribution focus on a single distribution center^10^. For the logistics distribution of multiple distribution centers in most researches, each distribution center is responsible for the customers in one region^11^. Unfortunately, this method is not applicable to the distribution problem due to the different inventory structure of each distribution center for industrial products, the influence of the logistics distribution radius of industrial products, and the transfer costs imposed by the legal permit system. In addition, some researchers have simplified the vehicle routing problem of multiple distribution centers to the vehicle path problem of a single distribution center^12^. Sadati et al. studied the trilevel r-interdiction selective multi-center vehicle routing problem (3LRI-SMDVRP)^13^. When Xiang Yang et al. studied the logistics distribution problem of multiple distribution centers, they established the corresponding objective function, but only minimized the distribution cost without involving other dimensions such as distribution time^14^. La Vega et al. studied the logistics distribution problem of a fleet of vehicles with the same type without considering the different models of contracted carriers^15^. Z. Su et al. studied the heterogeneous vehicle logistics distribution problem based on the parallel heuristic algorithm, but it does not involve the logistics distribution of multiple distribution centers^16^.

The multi-center VRP problem was studied in literature^17^. However, similar to literature^12^, it only establishes the single objective function of the lowest distribution cost, and does not consider the two objectives of vehicle utilization and distribution duration, nor does it consider the constraints of different types of vehicles. Srivastava et al. studied the multi-objective, single-center, single-type vehicle routing problem, and focused on the two objectives of distribution cost and distribution time^18^. Similarly, this work did not consider the vehicle utilization. On this basis, literature^19^ studies the path planning problem of vehicles with multiple objectives, single centers and multiple models. From the above work, it can be concluded that the current work on VRP has not established the corresponding mathematical model with the goal of minimum distribution cost, minimum distribution time and maximum utilization of distribution vehicles. There is no work to study the VRP problem of multi-target, multi-center and different types of vehicles at the same time. In addition, the above research is mostly based on some assumptions, such as the assumption that as long as there are goods vehicles, they will be delivered. This is not true in reality. The distribution company will deliver only after the loading rate of vehicles reaches a certain threshold.

### Algorithm

Most of the studies in the literature on algorithms for solving VPR problems are exact and heuristic algorithms^5^. Exact algorithms struggle to give effective solutions when the objective function and constraints are complex. Heuristic algorithms^20^ are widely used because of their better parallelism and low requirements on the characteristics of the objective function^21^. Montes et al. and Onieva et al. studied the optimization of logistics distribution paths based on evolutionary strategies^22,23^. Further, Peng Jiang et al. developed an evolutionary multi-objective algorithm^24^ to reduce the risk of dangerous goods transportation, based on which Z. Zhang et al. developed a multi-objective local search (MOLS) algorithm to avoid the algorithm from falling into local optima during the operation^25^. To solve the large-scale vehicle routing problem quickly and improve the algorithm's convergence accuracy, Y. Zhou et al. introduced a weight-space partitioning strategy and proposed a decomposition-based local search algorithm^26^. In addition, algorithms such as Ant Colony Optimization^27–29^, Genetic Algorithm^30,31^, and Particle Swarm Optimization^32–34^ are also widely used in the field of logistics and distribution routing optimization. In the process of algorithm improvement, all aforementioned algorithms aim to improve the convergence speed and shorten the search time.

The basic Group Search Optimizer (GSO) algorithm has been widely used in optimization problems since it was proposed in 2006^35,36^. Laithadualigah et al. successfully applied GSO algorithm to function selection (FS) problem in machine learning field, and studied the application of improved GSO search strategy in multi-objective optimization problem^37^. Further, Hamidteimourzadeh et al. established a single objective mathematical model with the goal of reducing the total loss of the distribution system. In order to make the GSO algorithm have better performance in solving such problems, they improved the GSO algorithm according to the problem characteristics^38^. Literature^39^ also introduced the mechanism of intraspecific competition (IC) and the searching strategy of Lévy walk (LW) into the basic GSO algorithm to improve the performance of the GSO algorithm. It can be seen that GSO algorithm and its improved algorithm have been widely used in optimization problems, but it is worth pointing out that because of the characteristics of the search strategy composed of discoverer, follower and wanderer in GSO algorithm, only the discoverer can change the search direction, and its ability to jump out of the local extreme value is not strong in the limited solution space. In addition, no relevant researchers have applied GSO algorithm to VRP problems and variants of VRP problems. According to the characteristics of GSO algorithm and based on the characteristics of biological life cycle, an improved life-cycle swarm optimization (ILSO) algorithm is designed to solve the model.

### Problem extension

With the further exploration of VRP by researchers, many variants of VRP have been developed at this stage. For example, the traditional VRP problem is improved, and the green VRP problem is generated with the goal of reducing carbon emissions^40^; Based on the background of emergency rescue after the disaster, the humanitarian logistics problems^41^. In addition, VRP is also applied to other fields. For example, Fanjul-Peyro et al. studied the machine scheduling problem in the manufacturing industry, improved the traditional VRP problem, and established a linear programming model for the machine scheduling problem^42^. Literature^43^ also improves the traditional VRP problem and applies it to the berth allocation and crane allocation of the terminal.

## Modeling the distribution path problem of heterogeneous vehicles for industrial products in multiple-depot with time windows

### Problem description

A industry company has multiple distribution centers in a certain area and multiple commercial client companies in several other areas. The statutory transportation permit system for the transportation of industrial products imposes the following limitations:

1. Each order of a commercial customer company must correspond to a time-effective shipping permit issued by the administrative department, and the order must be delivered within the validity period of the shipping permit.
2. Orders cannot be shipped by the way of cross-warehouse (Cross-warehouse or cross-docking refers to loading some goods from one distribution center and then loading them in another distribution center as shown in Fig. 1, which is not allowed.). When the inventory structure of the distribution center does not match the order demand, the inventory structure can be adjusted by transferring to the central warehouse to match demand and inventory.
3. A truck can carry more than one order, but each order can only be shipped by one carrier vehicle and cannot be shipped in separate vehicles for the same order.

Assume that each distribution center has enough industrial products of each model. However, due to the different warehouse models and inventory structures of various distribution centers, their outgoing capabilities are also different. Assume that the stacking gap is not considered in the consideration of the maximum load capacity, the default is no gap, different specifications of cargo packaging are the same. The unloading time is the same at each commercial customer company: three hours. Industry enterprises stipulate that when the no-load rate of each vehicle is less than 5%, a subsidy of 40 China Yuan (*CNY*) per vehicle trip is provided.

### Symbol description

Table 1 gives the symbol description of part of the model as follows.

**Table 1:** Symbol description of part of the model.

Symbol	Meaning
$G$	Distribution network,$G = \left( {V,A} \right)$
$V$	Point set,$V = U \cup J$
$A$	Arc set,$A = \left\{ {\left( {i,j} \right)\left| {i,j \in V,i \ne j} \right.} \right\}$
$U$	Distribution center collection,$U = \left\{ {1,2, \cdot \cdot \cdot ,u} \right\}$
$J$	Business customer company collection, $J = \left\{ {1,2,3, \cdot \cdot \cdot ,j} \right\}$
$L_{uj}$	The distance from the $u$ distribution center to the $j$ commercial customer company,$\forall L_{uj} \in A$
$L_{jj^{\prime}}$	The distance from the $j$ to the $j^{\prime}$ commercial client company,$\forall L_{jj^{\prime}} \in A$
$M$	Model collection of contracted carriers,$M{ = }\left\{ {{1, 2,} \cdot \cdot \cdot { ,}m} \right\}$
$X$	Contracted carrier vehicle number,$X = \left\{ {1,2, \cdot \cdot \cdot ,x} \right\}$
$xm$	The model of vehicle $x$ is $m$
$\omega_{j}$	Tonnage of the order of the $j$ commercial customer company
$\omega_{xmj}$	Tonnage of cargo delivered by vehicle $x$ to the $j - th$ commercial customer company
$\omega_{xmu}$	The tonnage of cargo loaded by vehicle $x$ at the $u - th$ distribution center
$t_{xmu}$	Pick-up time of vehicle $x$ at the $u - th$ distribution center
$t_{xmj}$	The storage time of vehicle $x$ in the $j - th$ commercial customer company ($t_{cmj}$ = 3)
$T_{xm}^{uj}$	The time for vehicle $x$ from the $u - th$ distribution center to the $j - th$ commercial customer company
$T_{xm}^{jj^{\prime}}$	The time for vehicle $x$ from the $j - th$ to the $j^{\prime} - th$ commercial client company for vehicle
$V_{u}$	Shipment speed of the $u$ distribution center
$P_{x}$	Indicates the total cost of vehicle $x$ in the transportation process
$T_{x}$	Indicates the total time of vehicle $x$ in the transportation process
$v_{m}$	Average speed of vehicles of model $m$
$W_{u\max }$	Maximum daily shipment weight of distribution center $u$
$\beta_{xmj}$	Decision variable, which indicates whether vehicle $x$ distributes to commercial customer company $j$,$\beta_{xmj} \in \left\{ {0 \, 1} \right\}$
$\alpha_{xmu}$	Decision variable, which indicates whether vehicle $x$ starts from distribution center $u$,$\alpha_{xmu} \in \left\{ {0 \, 1} \right\}$
$\xi_{xm}$	Decision variable, $\xi_{xm} \in \left\{ {0 \, 1} \right\}$, which indicates whether the loading rate ($\omega_{cmu} /\omega_{c\max }$) of goods loaded by vehicle $x$ is greater than 95%
$y_{j,j^{\prime}}^{xm}$	Decision variable, which indicates whether vehicle $x$ directly arrives at commercial customer company $j^{\prime}$ from commercial customer company $j$ during distribution,$y_{j,j^{\prime}}^{xm} \in \left\{ {0,1} \right\}$

### Mathematical model

Molina et al. established an adaptation degree function with minimum transportation cost and maximum service customers based on the heterogeneous vehicle path problem with hard time windows, which is valuable for the heterogeneous vehicle scheduling problem^44^. But its shortcoming is that it sets the transportation cost as a secondary optimization objective and does not consider the loading rate of the vehicle. Under this model, the loading rate of the vehicle is often too low, wasting resources and decreasing efficiency. In addition, there are also some studies^2,3^ in which the shortest total transportation mileage is the objective. They do not consider the vital influence of transportation volume. For example, when the aim is to minimize the transportation mileage, minimizing the freight expense (fuel consumption) when the vehicle is empty is neglected, leading to incomplete optimization and other problems. Therefore, this paper further improves the mathematical model established in document^41^, and builds the objective functions for shortest transportation time, lowest transportation cost, and most customers served by a single vehicle. Taking multiple objectives into account ensures the lowest transportation cost and shortest distribution time, improves the vehicle loading rate as much as possible, avoids wasting resources, and optimizes transportation routes and vehicle allocation. These improvements all contribute to improved distribution of industrial products.

Calculation rules for logistics, distribution, and transportation costs of industrial products are as follows:

1 $$ P_{actual} = \eta \times \omega \times L $$

where $P_{actual}$ represents the actual freight, $\eta$ represents the unit price of transportation in $CNY/\left( {Ton \times km} \right)$; $\omega$ is the weight of the cargo transported in $Ton$; $L$ is the transportation distance in $km$.

Therefore, this paper aims to minimize the transportation time, minimize the transportation cost, and maximize the average number of customers distributed by each vehicle. The model of the distribution route problem of heterogeneous vehicles with time windows for products in multiple distribution centers is as follows:

2 $$ \min f = \sum\limits_{x \in X} {P_{x} } \times \sum\limits_{x \in X} {T_{x} \times } \frac{{\sum\limits_{u \in U} {\sum\limits_{x \in X} {\alpha_{xmu} } } }}{{j_{\max } }} $$

where $j_{\max }$ is the number of commercial client companies.

3 $$ \begin{gathered} P_{x} = \eta \times \sum\limits_{j \in J} {\left[ {(\omega_{xmu} - \sum\limits_{j = 1}^{H} {\omega_{xmj} } ) \times L_{jj^{\prime}} } \right]} - 40 \times \sum\limits_{x \in X} {\xi_{xm} } \hfill \\ = \eta \times \sum\limits_{j \in J} {\left[ {(\sum\limits_{j \in J} {\omega_{xmj} } - \sum\limits_{j = 1}^{H} {\omega_{xmj} } ) \times L_{jj^{\prime}} } \right]} - 40 \times \sum\limits_{x \in X} {\xi_{xm} } \hfill \\ \end{gathered} $$

where formula (3) is the calculation formula for the total cost of vehicle $x$ in the transportation process. $\sum\limits_{j = 1}^{H} {\omega_{xmj} }$ is the weight of industrial products of commercial customer companies that have been delivered by vehicle $x$ before delivery to customer $j^{\prime}$. Where, $H$ is the number of commercial customer companies that vehicle $x$ has delivered before delivering to customer $j^{\prime}$,$j^{\prime}$ is the customer $\forall H \in J$, $\forall j^{\prime} \in J$. When $\sum\limits_{j = 1}^{H} {\omega_{xmj} } = 0$, $L_{jj^{\prime}} = L_{uj}$.

The total time $T_{x}$ of a vehicle during transportation is defined as follows.

4 $$ T_{x} = t_{xmu} + T_{xm}^{uj} + \sum\limits_{\begin{subarray}{l} j,j^{\prime} \in J \\ \, j \ne j^{\prime} \end{subarray} } {\left( {T_{xm}^{jj^{\prime}} \times x_{j,j^{\prime}}^{xm} } \right)} + \sum\limits_{\forall j \in J} {\left( {\overline{t}_{xmj} \times \beta_{xmj} } \right)} $$

where $\overline{t}_{xmj}$ is the warehousing time of vehicle $x$ in the $j$*-th* commercial customer company, which is easy to know from the assumption $\overline{t}_{xmj} = t_{xmj} = 3h$.The calculation methods of $T_{xm}^{uj}$, $T_{xm}^{jj^{\prime}}$ and $t_{xmu}$ are respectively shown in formulas (5), (6) and (7).

5 $$ T_{xm}^{uj} = \frac{{L_{uj} }}{{v_{m} }},\; \, \forall x \in X;\;\forall u \in U;\;\forall j \in J;\;\forall m \in M $$

6 $$ T_{xm}^{jj^{\prime}} = \frac{{L_{jj^{\prime}} }}{{v_{m} }}{, }\forall x \in X;\forall m \in M;\forall j,j^{\prime} \in J;j \ne j^{\prime} $$

7 $$ t_{xmu} = \frac{{\omega_{xmu} }}{{V_{u} }}, \, \forall x \in X;\forall u \in U;\forall m \in M $$

The constraints of this article are shown in formulas (8)-(18).

s.t.

8 $$ \omega_{m\min } \le \omega_{xmu} \le \omega_{m\max } {, }\forall x \in X;\forall u \in U;\forall m \in M $$

9 $$ \, \omega_{xmu} = \sum\limits_{j \in J} {\omega_{xmj} \times \beta_{xmj} ,} \, \forall x \in X;\forall u \in U;\forall m \in M $$

10 $$ \sum\limits_{u \in U} {\omega_{xmu} } \le W_{u\max } {, }\forall x \in X;\forall m \in M $$

11 $$ \sum\limits_{x \in X} {\alpha_{xmj} } = 1, \, \forall m \in M;\forall j \in J $$

12 $$ \sum\limits_{j \in J} {\beta_{xmj} } \le 3, \, \forall m \in M;\forall x \in X $$

13 $$ T_{j} \ge t_{xmu} + T_{xm}^{uj} + \sum\limits_{\begin{subarray}{l} j,j^{\prime} \in J \\ \, j \ne j^{\prime} \end{subarray} } {T_{xm}^{jj^{\prime}} } + \sum\limits_{j \in J} {\overline{t}_{xmj} \times \beta_{xmj} } ,\forall x \in X;\forall u \in U;\forall m \in M $$

14 $$ \sum\limits_{x \in X} {\alpha_{xmu} } \le \delta_{m\max } {, }\forall m \in M;\forall u \in U $$

15 $$ \sum\limits_{x \in X} {\beta_{xmj} } = 1, \, \forall m \in M;\forall j \in J $$

16 $$ \sum\limits_{x \in X} {\sum\limits_{m \in M} {\sum\limits_{j \in J} {\beta_{xmj} } } } = n $$

17 $$ \sum\limits_{x \in X} {y_{j,u}^{xm} } = 1, \, \forall \, j \in J;\forall u \in U;\forall m \in M $$

18 $$ \sum\limits_{\begin{subarray}{l} j \in J \\ j \ne j^{\prime} \end{subarray} } {y_{j,j^{\prime}}^{xm} = \sum\limits_{\begin{subarray}{l} k,j^{\prime} \in J \\ \, k \ne j^{\prime} \end{subarray} } {y_{j^{\prime},k}^{xm} } } {, }\forall x \in C;\forall m \in M $$

where formulas (8) and (9) constrain the upper and lower limit of loading of vehicle $x$. Formula (10) is the daily shipment limit of the distribution center $u$, which is determined by the warehouse structure, staff, and working hours. Formulas (11), (12), and (13) represent the limitation of the statutory transportation permit system. Formula (11) indicates that the vehicle cannot be loaded cross-warehouse; Formula (12) means that a delivery vehicle visits no more than three municipal power supply bureaus; Formula (13) enforces completion of the delivery task within the specified time. Formula (14) indicates that the vehicle type performing the delivery task does not exceed its upper limit. Formula (15) indicates that a customer can be served only once. Formula (16) is the sum of the customers served by each vehicle equals the total number of customers constraint. Formula(17) restricts the conservation of the in and out flow of vehicles in the distribution center, which means that vehicles can only directly reach one customer point from a distribution center. Formula (18) ensure the balance of the flow of vehicles entering and leaving the customer company in the service process.

## Model solution based on ILSO algorithm

The basic Group Search Optimizer (GSO) algorithm has been widely used in optimization problems since it was proposed in 2006^32–34^. However, when solving a problem with strong constraints, the optimal solution produced may no longer meet the requirements, and many local optimal solutions may also be produced. In addition, traditional mathematical optimization methods or some exact methods start from a single point in the search space and determine the next point through specific conversion rules. Compared with swarm intelligence optimization algorithms, their parallelism is lower. Therefore, this paper adopts the Life-cycle Swarm Optimization (LSO) algorithm, which is based on the life cycle characteristics of organisms and varies the number of populations in the algorithm. Further, we innovatively use the logistic population prediction model to predict the number of individuals in the control population and dynamically adjust the number of individuals in the population. This method simulates the natural evolution process of the population in the biological world. By making the population individuals more diverse, convergence happens faster in the optimization process. One can also control the time cost of the algorithm and improve the solution speed. The LSO algorithm provides an excellent choice to solve the problem of cargo distribution. Moreover, the LSO algorithm represents the parameter set of the problem as an individual and runs in the form of code instead of solving the parameters themselves as in traditional optimization algorithms. Therefore, when a computer processes the complex logistics scheduling model, the algorithm in this paper has good operability.

The basic LSO algorithm simulates the main processes of the life cycle, including growth, development, reproduction, and death. The summary of the basic LSO algorithm is as follows:

1. Parameter initialization.
2. Initialize the population randomly.
3. Assess the fitness value.
4. Iterative update of population individuals:
  - Perform chaotic chemotaxis operation: use Logistic equation to perform a chaotic search on the basis of the best individual in the current population.
  - Perform assimilation or transposition operation: the assimilation operation makes the individual evolve toward the optimal individual position, and the transposition operation makes the individual search within the energy range of its own.
  - Perform a breeding operation: pair the individuals in the population in sequence and perform a single-point crossover operation.
  - Perform death operations: sort the population individuals linearly according to their fitness values, adjust the fitness values, and select individuals for better optimization using roulette.
  - Perform mutation operation: change the evolution direction of the population according to the mutation probability.
  - Update the global extremum: calculate the fitness of individuals in the current population and update the current optimal individuals.
5. If the preset iteration stop condition is reached, the optimal solution and its fitness value will be output; if not, return to step 4.

### Explanation of symbols involved in the ILSO algorithm

$X_{{\text{i}}}^{k}$: In the *k-th* generation, the transportation scheduling plan represented by the *i-th* individual;$N_{U}$: Number of distribution centers;$N_{M}$: Number of models;$N_{od}$: Number of orders;$N_{um}$: The number of vehicles with model m under the *u-th* distribution center;$X_{{\text{i}}}^{k} (j).u$: Delivery center number of the shipping order number $j$, $u = 1,2,3,4$;$X_{{\text{i}}}^{k} (j).m$: Model number of shipping order number $j$, $m = 1,2,3$;$X_{{\text{i}}}^{k} (j).\delta$: The vehicle number of the shipping order number $j$, $\delta = 1,2, \ldots ,N_{um}$;$T_{\max }$: Maximum number of population iterations.

### The application of ILSO algorithm in the model

When applying the algorithm to a specific model, it is necessary to design the specific structure of the solution according to the actual problem, so that the problem can be adapted to the algorithm. The ILSO is divided into different phases to optimize different aspects of the problem at hand, just like how individuals go through different stages in their life cycle. And it draws on the characteristics of the biological life cycle: the number of individuals in the biological population is constantly changing, which conforms to the logistic population growth model. The ILSO algorithm also incorporates reproduction and mutation, which are biological processes that help population adapt and evolve over time. We also adopt multi-point crossover instead of single-point crossover to breed offspring individuals, and add roulette strategy when eliminating individuals. Based on this, we proposed the ILSO algorithm.

**Figure 2:**
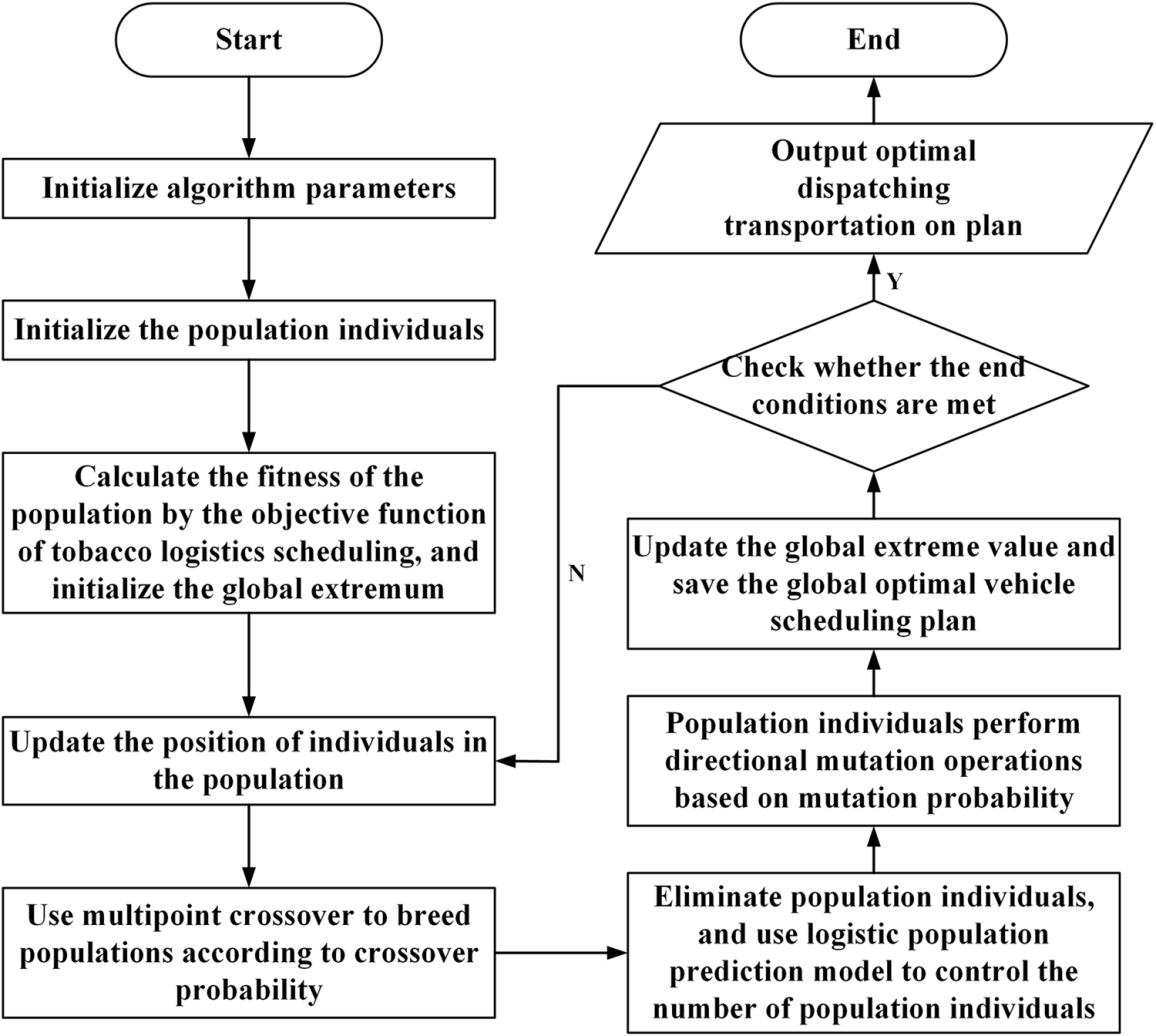
Algorithm flow chart.

The algorithm flow chart is shown in Fig. 2.

#### Representation of the solution

In the LSO algorithm, the position of an individual represents a solution to the problem, that is, a transportation scheduling plan. Due to the complex and complementary constraints of the model in this paper, the coding scheme must be intelligently designed, or it will be difficult to optimize the problem. But after modeling the problem reasonably and expressing the solution structure of the problem with an appropriate coding scheme, the algorithm proposed in this paper is suitable for most logistics scheduling optimization problems and has good adaptability.

**Figure 3:**
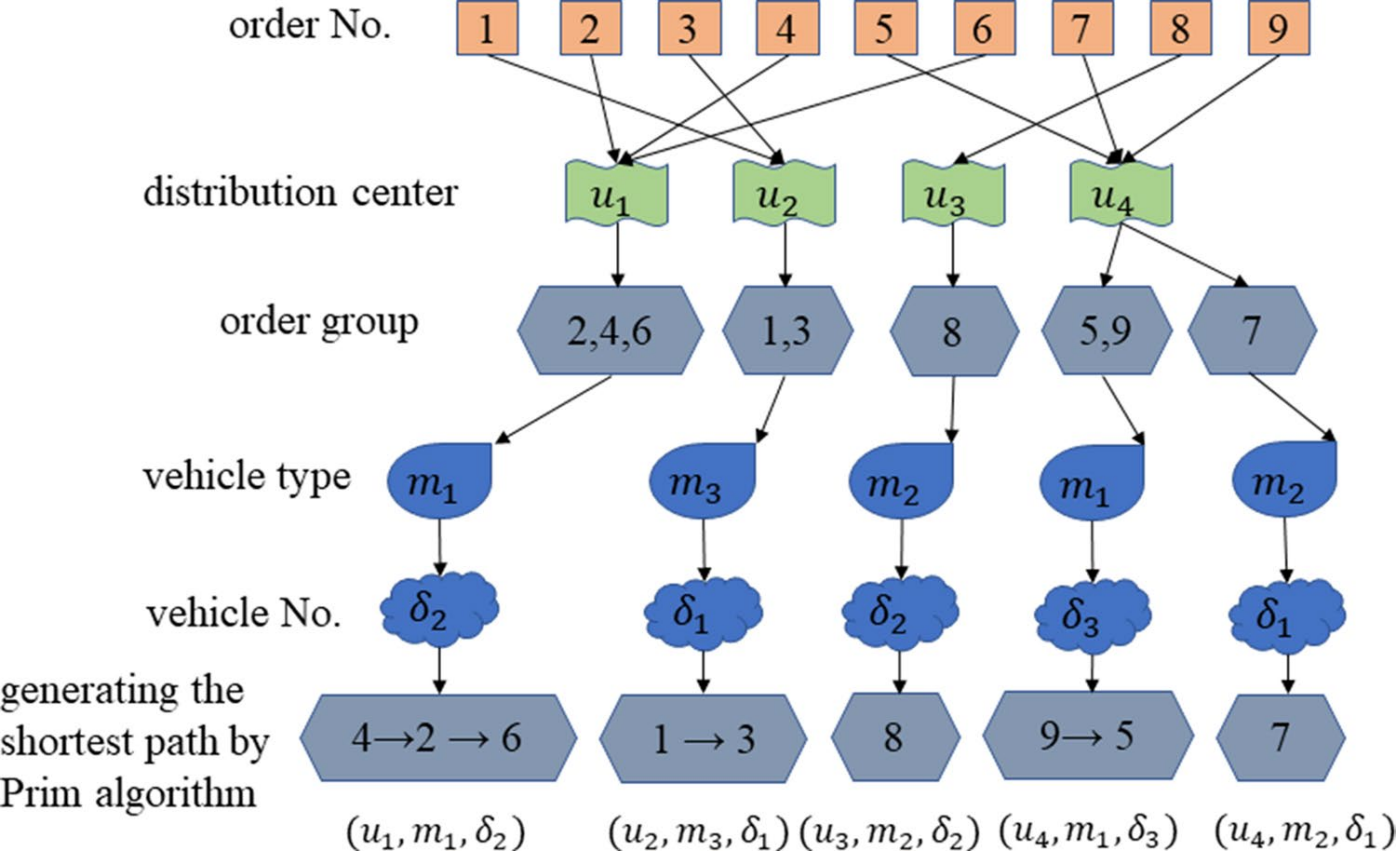
Schematic diagram of forming a dispatching transportation plan.

Based on this model, for a transportation scheduling scheme $u$, the distribution center $g$ accepts the order group $m$ and distributes it to the $\delta$-*th* vehicle of the model. The order group $m$ consists of 1–3 orders, which is a non-empty subset of the batch of orders, and the intersection of all order groups in a transportation scheduling plan is an empty set. The allocation of these orders is the multi-objective problem to be solved in this paper, including transportation time, transportation costs, and the completeness of order allocation. The algorithms need to balance the three of them to obtain the optimal objective function value. To make the delivery distance as short as possible, the delivery order of each order in the order group is determined by the Prim algorithm. It can be seen that the transportation scheduling plan of an order is determined by $(u,m,\delta )$ pair. The schematic diagram of the above-mentioned dispatching transportation process is shown in Fig. 3. The individual coding adopts the form of a structure array.

#### Initialization of the population

Due to the constraints of the upper and lower limits of vehicle loading and the number of orders, the allocation of orders is restricted. When the number of orders increases, the schemes that meet all constraints will drastically decrease, which is not conducive to the optimization of the algorithm. First, divide a batch of orders into several order groups, and filter the order groups that meet the upper and lower limits of vehicle loading and the number of orders. This initial optimization of the population individuals avoids the situation where the fitness of a large number of individuals in the population is extremely low.

Based on the above discussion, the population initialization steps are as follows:

1. Initialize the $X \cdot u$, $X \cdot m$ of each order according to the number of distribution centers $N_{U}$, the number of vehicle types $N_{M}$ , and the order grouping scheme.
2. Find the number of vehicles $X \cdot u$ with model $X.m$ under distribution center $N_{um}$ and $X \cdot \delta$ as a random integer of $\left[ {1,N_{um} } \right]$.
3. Repeat steps 1 and 2 to generate $pop_{\max }$ individuals.

**Figure 4:**
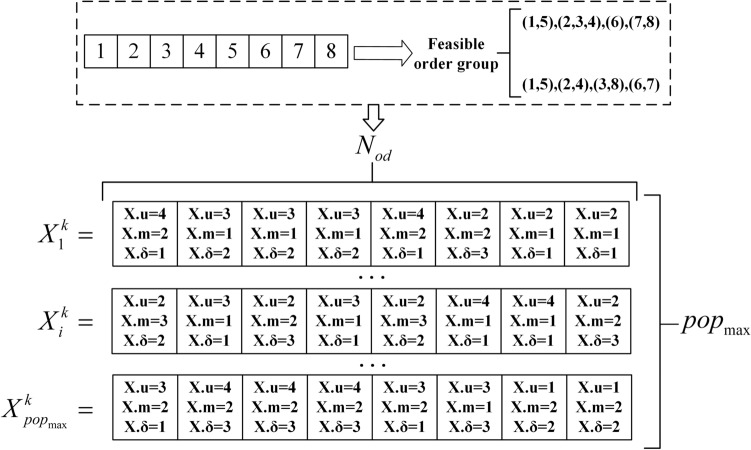
Schematic diagram of the generation of the initial population.

The schematic diagram of the initial population generation is shown in Fig. 4, which is the structure matrix $(pop_{\max } \times N_{od} )$.

After $pop_{\max }$ individuals are generated, the individual fitness value is calculated, and $N_{pop}^{1}$ individuals are selected as the primary population according to the roulette method.

#### Evaluation of individual fitness

The fitness value of the individual is calculated by the objective function. The corresponding law of the objective function is denoted as $f$, then for the individual $X_{{\text{i}}}^{k}$, its fitness value is $f(X_{i}^{k} )$. If the individual does not meet the constraints, assign a maximum value to its fitness.

#### Chaotic chemotaxis operation

The chaotic chemotaxis operation is a global search operation, which prevents the algorithm from falling into a local optimum due to insufficient search capabilities. The logistic equation is a typical chaotic system. The specific steps of using the logistic equation to perform chaotic operations on the optimal transportation scheduling plan $X_{{\text{g}}}^{k}$ are as follows:

- Map $X_{{\text{g}}}^{k}$ to the domain [0, 1] of the logistic equation to generate the initial chaotic variable $Z_{c}^{(1)}$, as shown in formula (19).
  19 $$ Z_{c}^{(1)} (j)\left\{ \begin{gathered} Z_{c}^{(1)} (j) \cdot u = \frac{{X_{{\text{g}}}^{k} (j) \cdot u - 1}}{{N_{U} - 1}} \hfill \\ Z_{c}^{(1)} (j) \cdot {\text{m}} = \frac{{X_{{\text{g}}}^{k} (j) \cdot m - 1}}{{N_{M} - 1}} \hfill \\ Z_{c}^{(1)} (j) \cdot \delta = \frac{{X_{{\text{g}}}^{k} (j) \cdot \delta - 1}}{{N_{um} - 1}} \hfill \\ \end{gathered} \right.\quad (j = 1,2, \ldots N_{od} ) $$
- 2. From the initial chaotic variable $Z_{c}^{(1)}$, a sequence of chaotic variables is generated iteratively according to the Logistic equation. The formula for the *(n* + *1)-th* chaotic variable ${\text{Z}}_{c}^{(n + 1)} n = {1}{\text{2,3}}...$ is shown in (20).
  20 $$ {\text{Z}}_{c}^{(n + 1)} {\text{ = Z}}_{c}^{(n)} \cdot (1 - {\text{Z}}_{c}^{(n)} ) $$
- 3. The chaotic variable sequence $$Z_{c}^{(n)}$$ is restored to the solution space by inverse mapping to obtain $$X_{{\text{c}}}^{(n)}$$$$(n = 1,2,3...)$$. Since the variables involved in this paper are integers, it needs to be rounded after restoration. The calculation formula for $$X_{c}^{(n)}$$ is
  21 $$ X_{c}^{(n)} (j)\left\{ \begin{gathered} X_{{\text{c}}}^{(n)} (j) \cdot u = \, \left[ {{\text{Z}}_{c}^{(n)} (j) \cdot u \times (N_{U} - 1) + 1} \right] \hfill \\ X_{{\text{c}}}^{(n)} (j) \cdot {\text{m}} = \, \left[ {{\text{Z}}_{c}^{(n)} (j) \cdot m \times (N_{M} - 1) + 1} \right] \hfill \\ X_{{\text{c}}}^{(n)} (j) \cdot \delta = \, \left[ {{\text{Z}}_{c}^{(n)} (j) \cdot \delta \times (N_{um} - 1) + 1} \right] \hfill \\ \end{gathered} \right., \, (j = 1,2, \ldots N_{od} ) $$
- 4. Calculate $$f(X_{c}^{(n)} )$$$$(n = 1,2,3 \ldots )$$, if $$f(X_{c}^{(n)} ) < f(X_{g}^{k} )$$, update the k*-th* generation optimal transportation scheduling plan, that is, let $$X_{{\text{g}}}^{k} = X_{c}^{(n)}$$.

#### Assimilation operation or transposition operation

Except for the best individual in the population, other individuals perform assimilation or transposition operations according to the selection probability $P_{select}$. The mathematical model that generates $X_{{\text{i}}}^{k + 1}$ from the assimilation operation of the transportation scheduling plan $X_{{\text{i}}}^{k}$ is:

22 $$ X_{i}^{k + 1} (j)\left\{ \begin{gathered} X_{i}^{k + 1} (j) \cdot u = \, [X_{i}^{k} (j) \cdot u + r_{1} \cdot (X_{g}^{k} (j) \cdot u - X_{i}^{k} (j) \cdot u)] \hfill \\ X_{i}^{k + 1} (j) \cdot m = \, [X_{i}^{k} (j) \cdot m + r_{1} \cdot (X_{g}^{k} (j) \cdot m - X_{i}^{k} (j) \cdot m)] \hfill \\ X_{i}^{k + 1} (j) \cdot \delta = \, [X_{i}^{k} (j) \cdot \delta + r_{1} \cdot (X_{g}^{k} (j) \cdot \delta - X_{i}^{k} (j) \cdot \delta )] \hfill \\ \end{gathered} \right.\quad (j = 1,2, \ldots N_{od} ) $$

The mathematical model of $X_{{\text{i}}}^{k}$ transposition operation to generate $X_{{\text{i}}}^{k + 1}$ is

23 $$ \left\{ {\begin{array}{*{20}l} {ub_{i}^{k} = \frac{{X_{p}^{k} }}{{X_{i}^{k} }} \cdot \Delta } \hfill \\ {lb_{i}^{k} = - ub_{i}^{k} } \hfill \\ {\varphi = r_{2} \left( {ub_{i}^{k} - lb_{i}^{k} } \right) + lb_{i}^{k} } \hfill \\ {X_{i}^{k + 1} = \left[ {X_{i}^{k} + \varphi } \right]} \hfill \\ \end{array} } \right. $$

$\Delta$ is the range of the entire solution space, the range between $ub_{i}^{k}$ and $lb_{i}^{k}$ is the maximum search range of $X_{i}^{k}$, $\varphi$ is called the transposition step length of $X_{i}^{k}$, and $r_{1}$ ,$r_{2}$ are uniformly distributed random numbers in (0, 1), $X_{p}^{k}$ is the optimal transportation scheduling plan for population in *k-th* generation.

#### Breeding operation and its improvement

**Figure 5:**
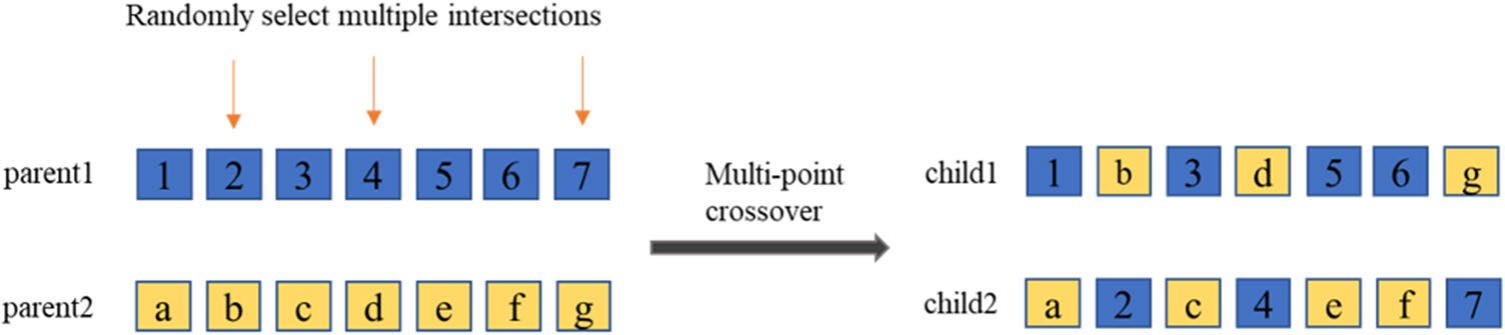
Schematic diagram of multi-point crossing.

The multipoint crossover was used for two individuals to reproduce offspring individuals according to the crossover probability $P_{cross}$. The multipoint crossover schematic is shown in Fig. 5. The reproduction operation in the original algorithm uses single-point crossover. Compared with multi-point crossover, single-point crossover is slower. In larger-scale problems, it will significantly increase the time cost of the algorithm, and the contribution to the diversity of offspring is less than that of multi-point crossover. Therefore, the algorithm in this paper adopts a multi-point crossover method.

#### Death operation and its improvement

The original algorithm uses a linear sorting method to adjust the population individuals according to their fitness values, and the adjusted fitness values are arranged in descending order, and then the individuals are selected by the roulette method. On this basis, this paper uses the logistic population growth model to control the number of individuals in each generation. The number of population individuals dynamically changes according to the logistic population growth model, which can shorten the algorithm's running time while ensuring the accuracy of convergence.

Assuming that the number of individuals in the k*-th* generation of the population is $N_{pop}^{k}$ , the number of individuals in the initial generation is $N_{pop}^{1}$, and the maximum population size set by the algorithm is $pop_{\max }$. When the number of individuals in the population reaches $\frac{{pop_{\max } }}{2}$ at $k = \frac{{T_{\max } }}{2}$, we can use the following equation of the logistic model to calculate the population growth rate $r$, and the number of individuals per generation:

24 $$ N_{pop}^{k} { = }\frac{{pop_{\max } }}{{1{\text{ + S}}e^{{ - r(k - k_{0} )}} }} $$

25 $$ S = \frac{{pop_{\max } - N_{pop}^{1} }}{{N_{pop}^{1} }} $$

In this paper, we use the elite strategy, i.e., the best individual is retained each time the roulette is performed, to prevent the best individual of the population from being eliminated, resulting in the algorithm's stability being affected.

The roulette algorithm uses the idea that each individual's probability is proportional to its fitness. The implementation steps are as follows:

- Calculate the probability of each individual being inherited into the k + 1 generation population from the fitness value $f(X_{i}^{k} )$ for each individual in the kth generation $P(X_{i}^{k} )$:
  26 $$ P(X_{i}^{k} ) = \frac{{f(X_{i}^{k} )}}{{\sum\nolimits_{j = 1}^{{N_{pop}^{k} }} {f(X_{j}^{k} )} }} $$
- Calculate the cumulative probability of each individual $q(X_{i}^{k} )$:
  27 $$ q(X_{i}^{k} ) = \sum\limits_{j = 1}^{i} {P(X_{i}^{k} )} $$
- Randomly generate random numbers $r_{3}$ in [0,1]. If $q(X_{i - 1}^{k} ) < r_{3} \le q(X_{i}^{k} )$, then select individual $X_{i}^{k}$.

#### Mutation operation

Mutation is used to promote the diversification of individuals in the population and prevent the algorithm from falling into a local optimum. The individual $X_{i}^{k}$ in the population performs a directional mutation operation according to the mutation probability $P_{mutate}$, and changes the asynchronous length to $L_{i}^{k}$:

28 $$ L_{i}^{k} (j)\left\{ \begin{gathered} L_{i}^{k} (j) \cdot u = \left[ {r_{3} \cdot ({\text{N}}_{U} - 1)} \right] \hfill \\ L_{i}^{k} (j) \cdot m = \left[ {r_{3} \cdot ({\text{N}}_{{\text{M}}} - 1)} \right] \hfill \\ L_{i}^{k} (j) \cdot \delta = \left[ {r_{3} \cdot ({\text{N}}_{um} - 1)} \right] \hfill \\ \end{gathered} \right.\quad (j = 1,2, \ldots N_{od} ) $$

Then $X_{i}^{k}$ is updated after mutation:

29 $$ X_{i}^{k} (j)\left\{ \begin{gathered} X_{i}^{k} (j) \cdot u = X_{i}^{k} (j) \cdot u + L_{i}^{k} (j) \cdot u \hfill \\ X_{i}^{k} (j) \cdot m = X_{i}^{k} (j) \cdot m + L_{i}^{k} (j) \cdot m \hfill \\ X_{i}^{k} (j) \cdot \delta = X_{i}^{k} (j) \cdot \delta + L_{i}^{k} (j) \cdot \delta \hfill \\ \end{gathered} \right.\quad (j = 1,2, \ldots N_{od} ) $$

## Results and analysis

### Results

In this section, we take an example of some pending orders for delivery on a particular day from China Southern Power Grid Co., Ltd,. The order information is shown in Table 2, and the distance matrix between each commercial customer company as well as the distribution center is shown in Table 3. S1, S2, S3 and S4 respectively represent Honghe, Huize, Kunming and Qujing distribution centers in Yunnan Province. This enterprise has four distribution centers, which are responsible for the distribution of industrial products for municipal power supply bureaus across the country and the information of each distribution center is shown in Table 4. All vehicles of each contracted carrier are one of three types of vehicles $M = \left\{ {1,2,3} \right\}$, and the vehicle information is detailed in Table 5. According to the data provided by China Southern Power Grid Co., we found that the unit price of transportation is $\mu = 0.25CNY{/}\left( {Ton \cdot km} \right)$ and a box of industrial products weighs 50* kg*. The location codes for each commercial customer company are shown in Table 6.

**Table 2:** Order information table.

Order city	Order weight (tons)	Valid time of transport permit (days)	Order city	Order weight (tons)	Valid time of transport permit (days)
C1	7	8	C21	8	10
C2	8	8	C22	6	10
C3	9	8	C23	10	10
C4	7	8	C24	6	10
C5	10	8	C25	11	10
C6	5	8	C26	9	11
C7	7	9	C27	5	11
C8	9	8	C28	6	12
C9	6	9	C29	6	12
C10	15	9	C30	6	11
C11	8	8	C31	8	11
C12	9	8	C32	8	11
C13	6	8	C33	7	11
C14	5	8	C34	7	13
C15	11	10	C35	5	13
C16	7	10	C36	10	13
C17	13	10	C37	11	8
C18	11	10	C38	11	8
C19	5	10	C39	9	8
C20	7	10	C40	5	8

**Table 3:** Distance matrix (Unit: *km*).

	C1	C2	…	C40	S1	S2	S3	S4
C1	0	96	…	775	2781	2612	2722	2423
C2	96	0	…	862	2759	2590	2702	2477
C3	394	388	…	766	2335	2186	2265	2037
C4	141	168	…	727	2529	2482	2562	2253
…	…	…	…	…	…	…	…	…
C30	1073	1034	…	1756	3854	3715	3887	3643
C31	918	910	…	1499	3848	3699	3732	3471
C32	1057	1043	…	1650	3968	3819	3919	3658
C40	775	862	…	0	2518	2479	2651	2316

**Table 4:** Daily shipment limit and shipment speed of each distribution center.

Storage section	S1	S2	S3	S4
Sunrise storage capacity (box)	7140	1200	10,600	17,000
Single shift speed (Box/hour)	2520	480	3780	2400

**Table 5:** Vehicle Information Parameter Table.

Vehicle model $\left( m \right)$	1	2	3
Loading limit $\left( {\omega_{m\min } } \right)$	13t	16t	15t
Loading limit $\left( {\omega_{m\max } } \right)$	24t	27t	25t
Average daily mileage of			
Vehicles (km)	648	648	648
Vehicle quantity	16	11	18

**Table 6:** Business customer company location code.

City	Symbol	City	Symbol	City	symbol
Beijing	C1	Dalian	C16	Songyuan	C31
Tianjin	C2	Benxi	C17	Harbin	C32
Handan	C3	Anshan	C18	Daqing	C33
Baoding	C4	Dandong	C19	Heihe	C34
Shijiazhuang	C5	Fushun	C20	Qiqihar	C35
Hengshui	C6	Tieling	C21	Shangzhi	C36
Qinhuangdao	C7	Panjin	C22	Ulanhot	C37
Cangzhou	C8	Jinzhou	C23	Erenhot	C38
Chengde	C9	Yingkou	C24	Hailar	C39
Zhangjiakou	C10	Fuxin	C25	Linhe	C40
Xingtai	C11	Changchun	C26	Honghe	S1
Langfang	C12	Siping	C27	Huize	S2
Shahe	C13	Tonghua	C28	Kunming	S3
Botou	C14	Baicheng	C29	Qujing	S4
Shenyang	C15	Dunhua	C30		

The parameters of the algorithm, such as the population size, are shown in Table 7. These parameters are the best parameters selected by multiple tests to solve the problem in this paper.

**Table 7:** Algorithm parameter table.

Algorithm	Parameter	Symbol	Value
PSO	Initial population	$pop_{new}$	807
	Maximum number of iterations	$T_{\max }$	60
	Learning factor	$c_{1}$	2
	Learning factor	$c_{2}$	2
	Inertia weight	$\omega ^{\prime}$	0.65
	Maximum particle velocity	$V_{MAX}$	1.2
	Particle minimum velocity	$V_{MIN}$	-1.2
ILSO	Initial population	$N_{pop}^{1}$	10
	Maximum population	$pop_{\max }$	807
	Maximum number of iterations	$T_{\max }$	60
	Probability of choice	$P_{select}$	0.8
	Crossover probability	$P_{cross}$	0.7
	Mutation probability	$P_{mutate}$	0.1
BA	Initial population	$pop_{\max }$	807
	Maximum number of iterations	$T_{\max }$	60
	Minimum frequency	$f_{\min }$	0
	Maximum frequency	$f_{\max }$	1
	Initial transmission frequency	$r_{o}$	0.7
	Constant	$\alpha$	0.9
	Constant	$\gamma$	0.9
WOA	Initial population	$pop_{new}$	807
	Maximum number of iterations	$T_{\max }$	60
GWO	Initial population	$pop_{new}$	807
	Maximum number of iterations	$T_{\max }$	60
MA	Initial population	$pop_{new}$	807
	Maximum number of iterations	$T_{\max }$	60
	Positive attraction constants	$a_{1}$	0.6
	Positive attraction constants	$a_{2}$	0.8
	The fixed visibility coefficient	$\beta$	0.2

**Figure 6:**
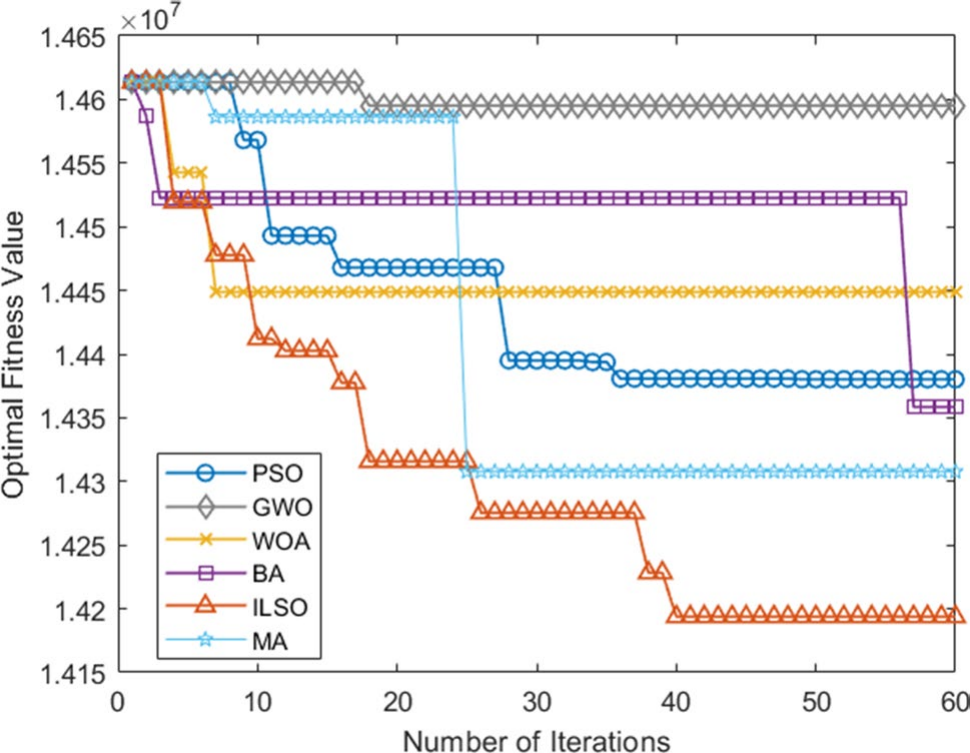
Convergence curve of the objective function in a certain run of five algorithms.

**Figure 7:**
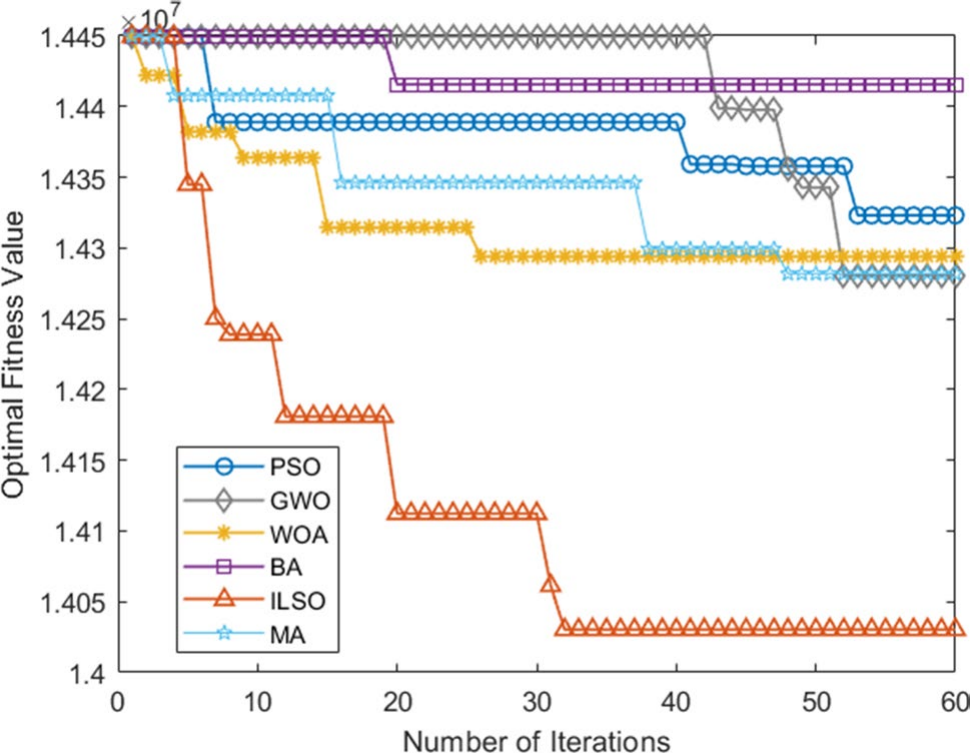
The convergence curve of the optimal objective function of each algorithm in the fifteen runs of the five algorithms.

**Figure 8:**
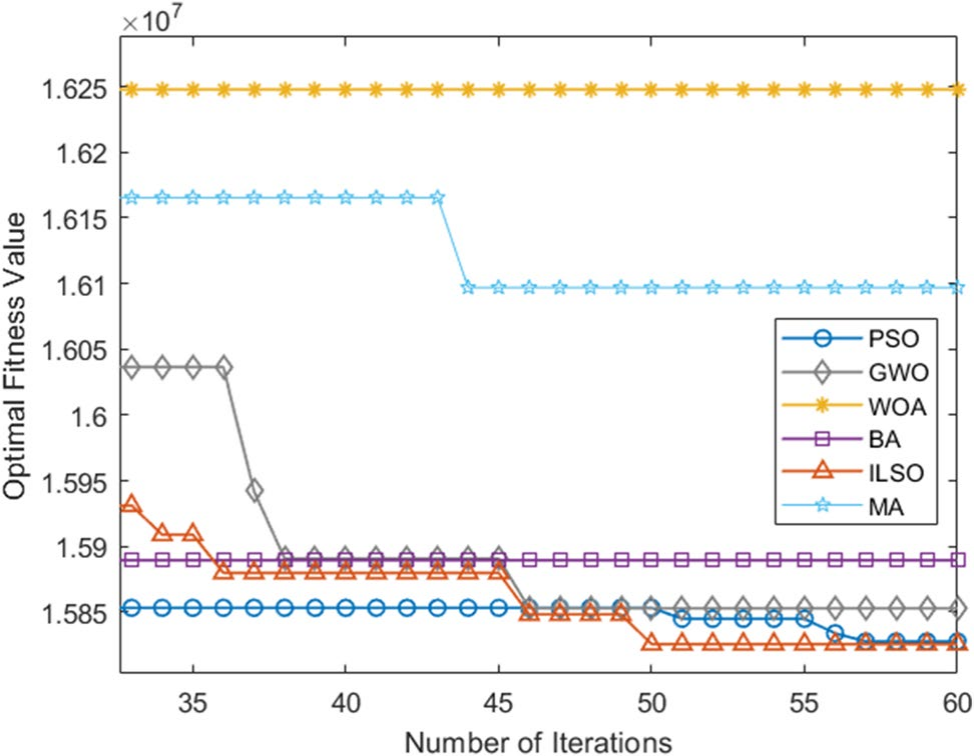
Convergence curve of the worst objective function of each algorithm in fifteen runs of five algorithms.

**Figure 9:**
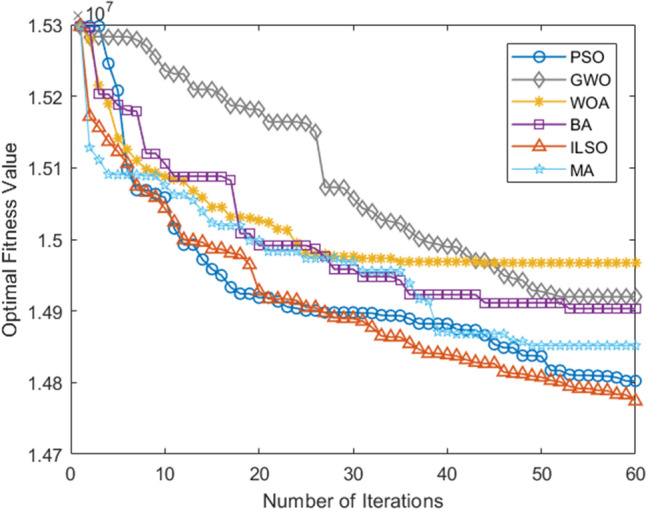
Average convergence curve of the objective function over fifteen runs of the five algorithms.

The algorithm programming tool in this paper was MATLAB R2017a, the operating system was Windows 10, the computer memory was 16G, and the CPU was Intel i7-8750H. For comparison, the developed algorithm was compared with five other biological heuristic algorithms including Bat Algorithm (BA), Particle Swarm Algorithm (PSO), Whale Algorithm (WOA), Gray Wolf Algorithm (GWO), Mayfly Algorithm (MA), and conducted fifteen simulation experiments in total. The results are as follows, including the planning results of the ILSO algorithm during the fifteen runs of each algorithm randomly selected (Table 8), the convergence curve of the five algorithms at that time (Fig. 6), and the comparison table of each dimension index (Table 9). The comparison chart of the optimal/worst convergence curves of the five algorithms running fifteen times (Figs. 7,8), the comparison of average convergence curves, and the comparison table of indicators (Fig. 9, Tables 10, 11).

**Table 8:** Randomly selected planning results for one of the fifteen runs of the ILSO algorithm.

Route	Specific route	Delivery model
1	S1 → C5 → C12 → S1	Model 1
2	S1 → C10 → C28 → S1	Model 3
3	S2 → C15 → C21 → S2	Model 1
4	S2 → C9 → C25 → C24 → S2	Model 1
5	S2 → C18 → C16 → C34 → S2	Model 2
6	S2 → C13 → C17 → C27 → S2	Model 2
7	S2 → C14 → C7 → C35 → S2	Model 3
8	S2 → C22 → C29 → C36 → S2	Model 3
9	S2 → C11 → C40 → C38 → S2	Model 3
10	S2 → C20 → C26 → C31 → S2	Model 3
11	S3 → C8 → C23 → S2	Model 3
12	S3 → C2 → C30 → C32 → S2	Model 3
13	S4 → C4 → C1 → C19 → S2	Model 1
14	S4 → C37 → C33 → S2	Model 2
15	S4 → C3 → C6 → C39 → S2	Model 3

**Table 9:** Comparison table of the metrics for each of the five algorithms for a given run.

Algorithm	Index				
	I1	I2	I3	I4	I5
PSO	436,281.83	53,593.22	5.86	14,380,209.98	3
GWO	439,886.08	53,993.22	5.90	14,595,135.93	2
WOA	437,686.33	53,698.22	5.87	14,448,746.91	1
BA	436,301.83	53,508.22	5.85	14,358,496.82	1
MA	436,387.26	53,564.22	5.89	14,307,749.80	2
ILSO	432,763.58	53,201.22	5.82	14,193,775.96	3

In Table 9, the definitions of individual indicators (I1, I2,…) are shown in Table 10. Noteworthily, objective function value is calculated by multiplying the time spent on each group of orders by the freight of this distance, and then sum them up, rather than simply multiplying the total freight by average transit time.

**Table 10:** In Table 9, the meaning represented by each indicator.

Contents		Contents	
Total freight (*CNY*)	I1	Objective function value	I4
Total route length (km)	I2	The number of vehicles with an idling rate of less than 5%	I5
Average transit time (days)	I3

**Table 11:** The index values of the five algorithms during fifteen runs.

Algorithm	Index		
	Optimal solution of objective function	Worst solution of objective	Average running time (seconds)
PSO	14,323,058	15,827,555	152.107
GWO	14,280,636	15,852,784	154.821
WOA	14,293,834	16,247,874	168.380
BA	14,415,635	15,889,506	155.153
MA	14,281,815	16,096,964	158.573
ILSO	14,030,245	15,825,517	104.959

**Figure 10:**
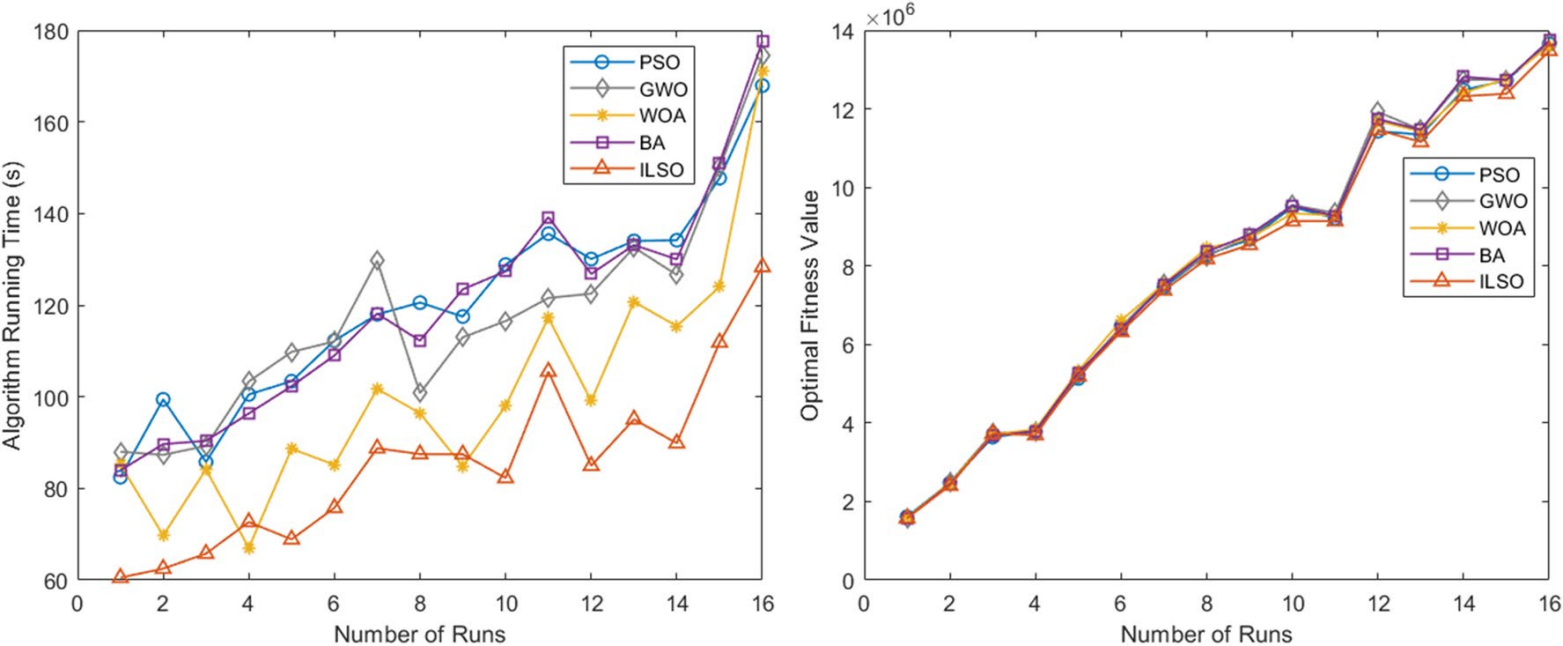
Effect of variation in the number of orders on the running time and optimal objective function of each algorithm.

A further experiment involved selecting the city name from the database and randomly generating a series of orders of different numbers corresponding to the valid time of the transportation permit. We conducted 16 random generation orders, and the order quantity increased from 15 to 30. This examined the stability and dynamic performance of the algorithm, and the effect of varying the number of orders on the running time of each algorithm and the optimal objective function. The results over sixteen runs are shown in Fig. 10.

Figure 10a shows the effect of the change in the number of orders on the running time of each algorithm. Figure 10b shows the effect of the change in the number of orders on the optimal objective function over the fifteen runs of each algorithm.

**Figure 11:**
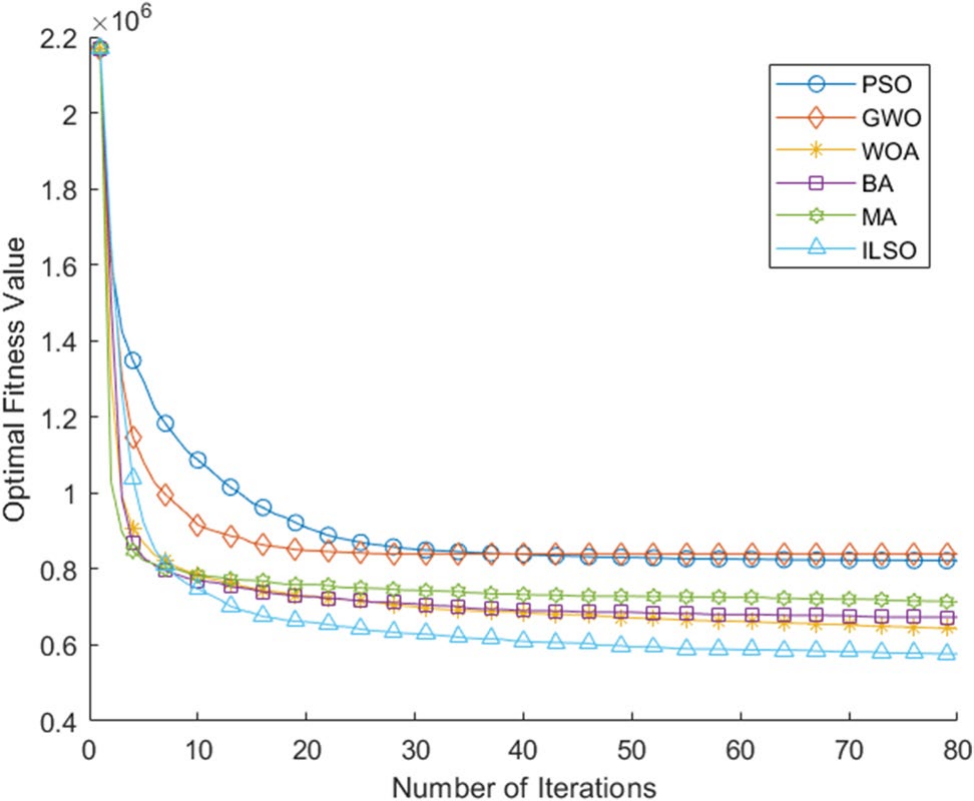
The average iteration curve of objective function value for Solomon's VRPTW benchmark test after 50 runs.

**Figure 12:**
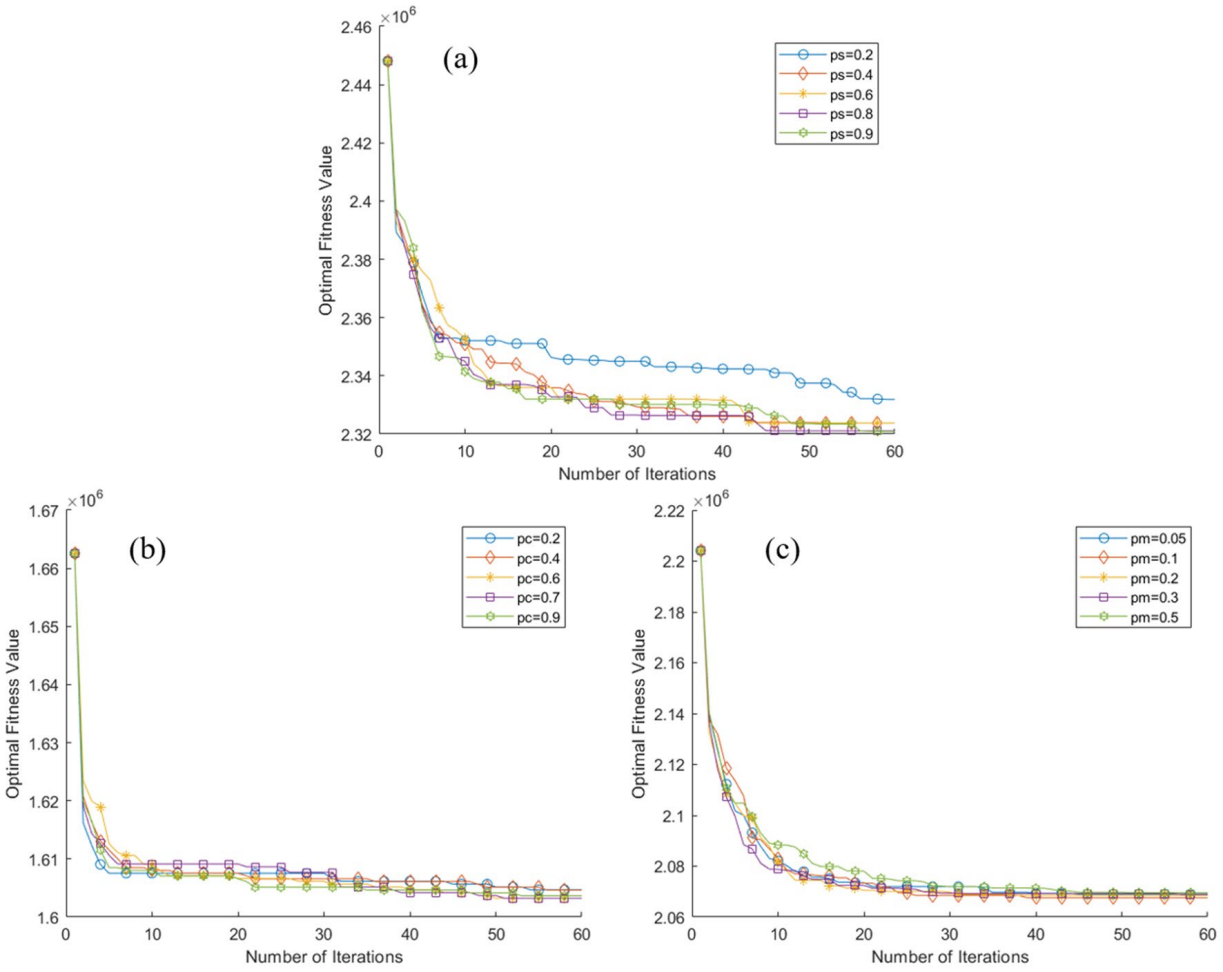
Parameter sensitivity analysis of ILSO algorithm.

In addition, in order to further study the stability and reliability of the algorithm, we also conducted the Solomon's benchmark test of vehicle routing problem with time window constraints and parameter sensitivity analysis of ILSO algorithm. The average iteration curve after 50 runs of each of the six algorithms is shown in Fig. 11. More detailed results are shown in Table 12. Parameter sensitivity analysis of ILSO algorithm is shown in Fig. 12. In Fig. 12, (a) ps in the figure represents probability of choice, (b) pc in the figure represents crossover probability, and (c) pm in the figure represents mutation probability.

**Table 12:** The index values of the five algorithms during fifteen runs.

Algorithm	Index				
	I6	I7	I8	I9	I10
PSO	820,534.94	23	3602.97	22	95
GWO	840,181.88	24	3634.01	25	100
WOA	628,406.96	24	3509.95	22	94
BA	667,769.95	25	3553.67	23	96
MA	710,109.54	24	3666.91	23	96
ILSO	567,334.37	24	3373.42	17	92

In Table 12, the definitions of individual indicators (I6, I7,…) are shown in Table 13.

**Table 13:** In Table 12, the meaning represented by each indicator.

Contents		Contents	
Objective function value	I6	Number of routes violating constraints	I9
Number of vehicles used	I7	Number of customers violating constraints	I10
Vehicle driving distance	I8		

**Figure 13:**
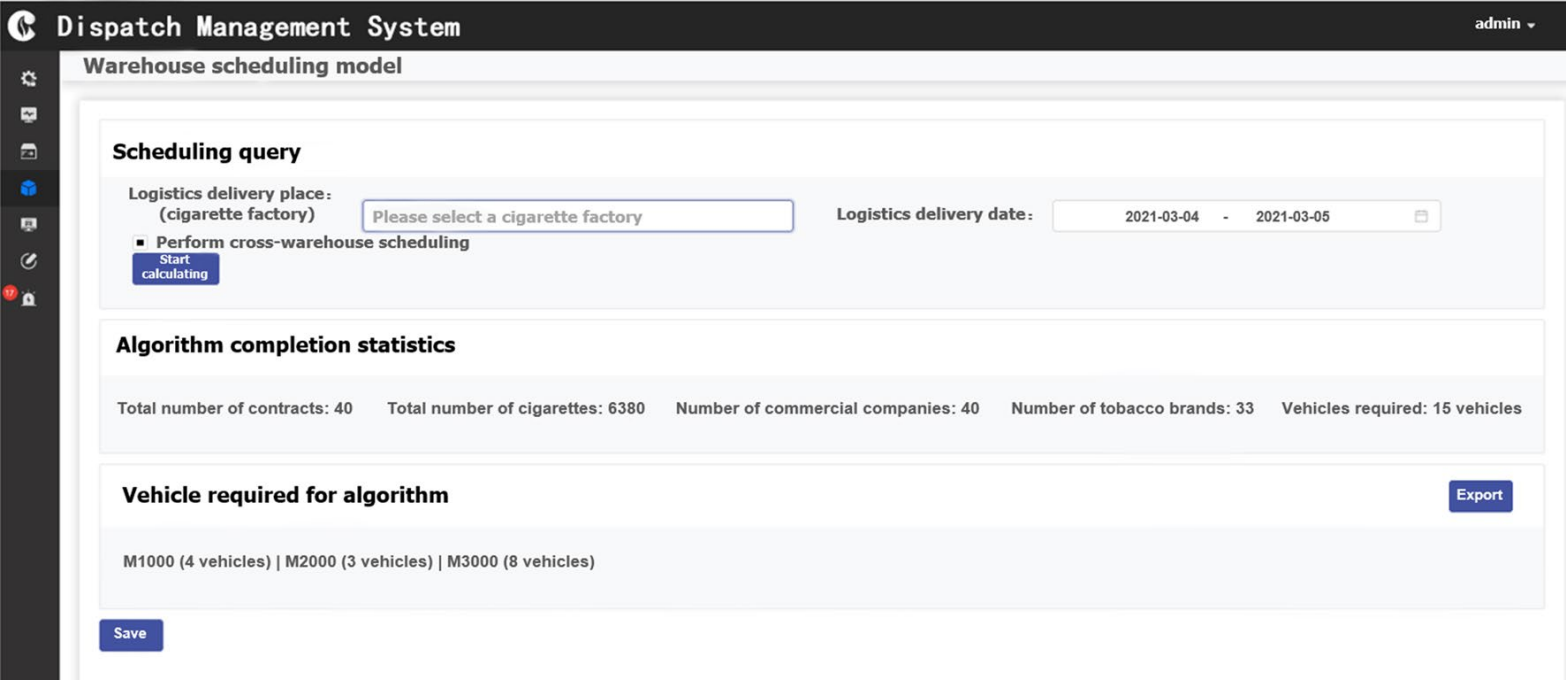
Display interface for ILSO algorithm calculation result details.

In the following, we show the developed model and the interface of the algorithm applied to the scheduling system of China Southern Power Grid Co., Ltd. In addition, a screenshot of the order details interfaces after the logistics distribution multi-objective optimization model and ILSO algorithm has been calculated for a certain day's order is exhibited. It includes the order quantity, the number of vehicles of the delivered customer company, and the required vehicle information, as shown in Fig. 13.

### Result analysis

The above simulation experimental results show that the ILSO algorithm proposed in this paper is very applicable to the strongly constrained problem of logistics distribution by multi-center heterogeneous vehicles under multidimensional constraints. Table 9 shows that when the corresponding parameters of each algorithm are the same, the total freight, total route length, average transportation time, objective function value, and the number of vehicles with an empty load rate of less than 5%, the ILSO algorithm is the best. ILSO algorithm reduces transportation cost by 0.8%, 1.6%, 1.1%, 0.8%, and 0.8% compared with PSO, GWO, WOA, BA and MA respectively. According to the survey, the Yunnan Power Grid Corporation's transfer costs counted between May 2019 and May 2020 is $1,480.371 \times 10^{4}$ *CNY*, which will enable the group to save at least $11.843 \times 10^{4}$ *CNY* per year on freight costs. In addition, Table 9 shows that the ILSO algorithm results in the shortest average transit time, which greatly improves the on-time delivery of industrial products. Figure 6 shows that the ILSO algorithm has the fastest convergence speed and highest convergence accuracy than the other five algorithms.

Furthermore, from the convergence curves of each algorithm's optimal, worst, and average objective function in the fifteen runs of the five algorithms (Figs. 7, 8, 9) and Table 10 can conclude that the ILSO algorithm is the best from the perspective of the optimal value, the worst value, and the average value. It can be seen from Table 10 that in the process of running the five algorithms fifteen times, the average running time of the ILSO algorithm is the shortest, only 104.9593 s. Compared with the other five algorithms, it saves 47.78%-60.42% of the calculation time, which provides a guarantee for the designed model and algorithm to solve larger orders. As seen in Fig. 10, each algorithm increases the corresponding running time as the number of orders increases, and it shows ILSO has better performance on running time. For Solomon's VRPTW benchmark test, it can be seen from Fig. 11 and Table 12 that ILSO has great advantages in the indicators of vehicle driving distance, number of routes violating constraints and number of customers violating constraints. Figure 12 shows after more than 50 iterations, as the parameters change, the objective function value has little impact, indicating that the 60 iterations set in this work are reasonable. We applied the model and ILSO algorithm in the paper to the actual logistics scheduling, and developed a set of logistics scheduling system. Figure 13 is the interface of the system, which can efficiently and reasonably conduct logistics scheduling.

## Conclusion

This research focuses on the logistics and distribution of industrial products. It focuses on the cross-regional heterogeneous vehicle scheduling mathematical model and algorithm design of multi-distribution centers and multi-municipal power supply bureaus. It develops an ILSO, making it the best in solving forty commercial client companies 6380 dan industrial products.

First, a mathematical model of cross-regional heterogeneous vehicle scheduling with multiple distribution centers and multiple municipal power supply bureaus was established, and the ILSO algorithm was further developed. Then, finally, by processing the pending orders of China Southern Power Grid Co., Ltd, one day. The overall results show that the ILSO algorithm has higher convergence speed, convergence accuracy, and lower computing time than the other five biological heuristic algorithms. Furthermore, compared with the other five algorithms, the results of this algorithm reduce transportation costs by 0.8%-1.6% and reduce computing time by 47.78%-60.42%. Moreover, the ILSO algorithm can solve large-scale examples in the shortest time. It can be effectively applied in the dispatching system developed by the group, effectively solving the multi-objective complex heterogeneous vehicle routing problem involving numerous constraints.

## Data Availability

The datasets generated and/or analyzed during the current study are not publicly available due to the confidentiality agreement signed between Kunming University of Science and Technology and China Southern Power Grid Co., Ltd, but are available from the corresponding author on reasonable request.
